# Chemical Diversity, Biological Activity, and Genetic Aspects of Three *Ocotea* Species from the Amazon

**DOI:** 10.3390/ijms18051081

**Published:** 2017-05-18

**Authors:** Joyce Kelly da Silva, Rafaela da Trindade, Edith Cibelle Moreira, José Guilherme S. Maia, Noura S. Dosoky, Rebecca S. Miller, Leland J. Cseke, William N. Setzer

**Affiliations:** 1 Programa de Pós-Graduação em Biotecnologia, Universidade Federal do Pará, 66075-900 Belém, Brazil; joycekellys@ufpa.br (J.K.d.S.); rcabral@ufpa.br (R.d.T.); 2 Instituto de Estudos em Saúde e Biológicas, Universidade Federal do Sul e Sudeste do Pará, 68501-970 Marabá, Brazil; cibelle@unifesspa.edu.br; 3 Programa de Pós-Graduação em Recursos Naturais da Amazônia, Universidade Federal do Oeste do Pará, 68035-110 Santarém, Brazil; gmaia@ufpa.br; 4 Department of Chemistry, University of Alabama in Huntsville, Huntsville, AL 35899, USA; nouradosoky@yahoo.com; 5 Department of Biological Sciences, University of Alabama in Huntsville, Huntsville, AL 35899, USA; rsm0024@uah.edu (R.S.M.); csekel@uah.edu (L.J.C.)

**Keywords:** Lauraceae, volatile compounds, terpenes, *matK* gene, phylogenetic analysis

## Abstract

*Ocotea* species present economic importance and biological activities attributed to their essential oils (EOs) and extracts. For this reason, various strategies have been developed for their conservation. The chemical compositions of the essential oils and *matK* DNA sequences of *O. caudata*, *O. cujumary*, and *O. caniculata* were subjected to comparison with data from *O. floribunda*, *O. veraguensis*, and *O. whitei*, previously reported. The multivariate analysis of chemical composition classified the EOs into two main clusters. Group I was characterized by the presence of α-pinene (9.8–22.5%) and β-pinene (9.7–21.3%) and it includes *O. caudata*, *O. whitei*, and *O. floribunda*. In group II, the oils of *O. cujumary* and *O. caniculata* showed high similarity due amounts of β-caryophyllene (22.2% and 18.9%, respectively). The EO of *O*. *veraguensis*, rich in *p*-cymene (19.8%), showed minor similarity among all samples. The oils displayed promising antimicrobial and cytotoxic activities against *Escherichia coli* (minimum inhibitory concentration (MIC) < 19.5 µg·mL^−1^) and MCF-7 cells (median inhibitory concentration (IC_50_) ≅ 65.0 µg·mL^−1^), respectively. The analysis of *matK* gene displayed a good correlation with the main class of chemical compounds present in the EOs. However, the *matK* gene data did not show correlation with specific compounds.

## 1. Introduction

The genus *Ocotea* is not a monophyletic group of the Lauraceae, which includes about 400 species occurring mostly in tropical and subtropical regions (Central and South America, the West Indies, and Africa) [1,2]. These species present alternate penninerved leaves; inflorescenses thyrsopaniculate to botryoid. Flowers are trimerous, bisexual, polygamous, or unisexual; tepals equal, rarely persistent on the rim; nine fertile stamens, the third whorl with glands; anthers with four loculos; receptacle very small and deeply tubular; in male flowers, rudimentary ovary to absent; fruit and cupule extremely variable in size and shape [1]. It is a very variable genus morphologically, being the largest genus in the Neotropics, with 170 species occurring in Brazil [3,4].

The economic importance of *Ocotea* species in the Amazon region has been related to numerous applications such as the use of their wood in lightweight construction and luxury furniture [5]. Phytochemical studies reported the occurrence of benzylisoquinoline alkaloids, neolignans, catechins from leaves and bark of *O. porosa* [6,7]; aporphine alkaloids from leaves of *O. macrophylla*; flavonols from *O. vellosiana*; and eudesmane sesquiterpenoids from *O. corymbosa* [8,9,10]. The volatile chemical profiles of *Ocotea* species are characterized by high concentrations of phenylpropanoids and terpenoids (hydrocarbons or oxygenated) [11,12].

Many studies have been reported on the biological activities of *Ocotea* metabolites: the alkaloid reticuline isolated from extract of *O. duckei* showed potent central nervous system depressant action [13]. (−)-Caaverine, a noraporphine alkaloid isolated from *O. lancifolia*, has shown high antiprotozoal activity against *Leishmania* and *Trypanosoma cruzi* parasites [14]. The chloroform fraction obtained from an extract of fruits of *O. puberula* and the alkaloid dicentrine displayed antinociceptive effects [15]. The butanolides isolated from roots of *O. macrocarpa* showed good cytotoxic activities against the A2780 ovarian cell line [16]. The flavonoids of *O. notata* showed antimycobacterial activity and ability to inhibit NO production by macrophages [17]. The essential oil of *O. quixos*, rich in *trans*-cinnamaldehyde and methyl cinnamate, showed anti-inflammatory properties in vitro and in vivo models [18]. Studies on the chemical characteristics of the species *O. caudata*, *O. cujumary*, and *O. caniculata* are rare and important because *Ocotea* species are classified as threatened to extinction by the Brazilian List and the risk of extinction is increased due the reduction of genetic variability [19,20] so knowledge of the genetic diversity is necessary for our understanding of the factors that determine essential oil quantity and quality in these economically important species [21].

## 2. Results and Discussion

### 2.1. Essential Oil Chemical Composition

The essential oil (EO) of *Ocotea* species provided different yields and the higher yields were found in the leaf EO for all samples (0.7–0.8%) (Table 1).

**Figure 1:**
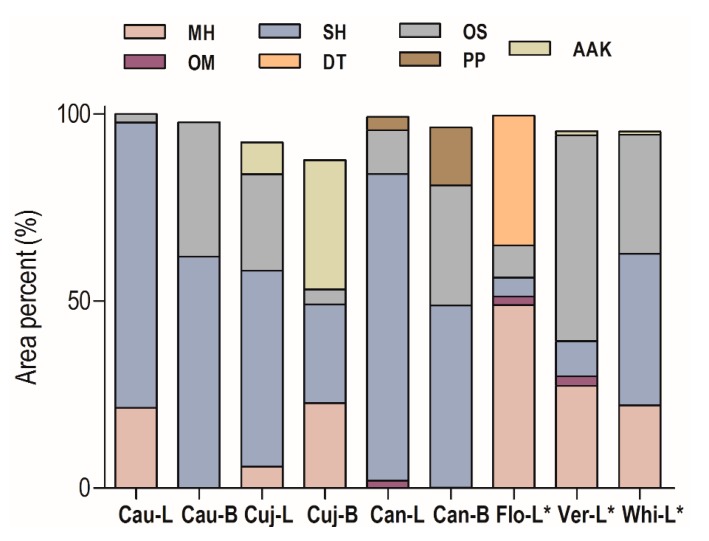
Distribution of compound classes in essential oils (Eos) of *Ocotea* species. Monoterpene hydrocarbons (**MH**), Oxygenated monoterpenoids (**OM**), Sesquiterpene hydrocarbons (**SH**), Oxygenated sesquiterpenoids (**OS**), Diterpenes (**DT**), Phenylpropanoids (**PP**), Alkanes, aldehydes, and ketones (**AAK**). *O. floribunda* leaves (**Flo-L**), *O. veraguensis* leaves (**Ver-L**), *O. whitei* leaves (**Whi-L**); * Literature data [22]. **Cau-L** (*O. caudata* leaves); **Cau-B** (*O. caudata* bark); **Cuj-L** (*O. cujimary* leaves); **Cuj-B** (*O. cujimary* bark); **Can-L** (*O. caniculata* leaves); **Can-B** (*O. caniculata* bark).

The most representative compounds class was sesquiterpene hydrocarbons (56.3–82.0%) in all samples from Caxianuã Forest (Figure 1). The *O. caudata* oils showed different chemical profiles in the tissues; in the EO from leaves (**Cau-L**) the monoterpene hydrocarbons (21.5%) were present, while in the branches (**Cau-B**) there was a high accumulation of oxygenated sesquiterpenoids (35.9%). The EO of *O. cujumary* leaves (**Cuj-L**) showed higher concentrations of oxygenated sesquiterpenoids (25.8%) than branches (**Cuj-B**, 4.0%), which showed significant amounts of monoterpene hydrocarbons (22.7%) and fatty acid derivatives (34.6%). The *O. caniculata* oils showed amounts of phenylpropanoids, especially in the branches (**Can-B**, 15.5%). Seventy volatile components were identified, comprising approximately 95.1% of the total composition of the oils (Table 2).

The main compounds identified in *O. caudata* oils were bicyclogermacrene (29.6%), germacrene D (19.9%), β-caryophyllene (9.6%), α-pinene (9.8%), and β-pinene (9.7%) in the leaves (**Cau-L**) and δ-cadinene (13.8%), germacrene D (8.9%), and α-muurulol (7.8%) in the branches (**Cau-B**). β-Caryophyllene, germacrene-D, α-pinene, and β-pinene were the principal common constituents of the EOs of leaves of *Ocotea floribunda*, *O. holdridgeana*, *O. meziana*, *O. sinuata*, *O. tonduzii*, *O. valeriana*, *O. veraguensis*, *O. whitei*, and two new undescribed *Ocotea* species [22].

In addition, these chemical compounds were identified in the leaves in other Lauraceae species. The EO of leaves of *Endlicheria arenosa* was dominated by bicyclogermacrene (42.2%), germacrene D (12.5%), and β-caryophyllene (10.1%) [23]. Similarly, the EO of *Nectandra leucantha* showed as the main compounds bicyclogermacrene (28.4%), germacrene A (7.3%), α-pinene (6.6%), and β-pinene (4.6%) [24]. The sesquiterpene δ-cadinene identified in the EO of branches was detected as the main compound in EOs of leaf and bark of *Beilschmiedia madang* (17.0% and 20.5%, respectively) and was associated with germacrene D in the oil of barks of *Beilschmiedia glabra* [25,26].

The EO of *O. cujumary* leaves (**Cuj-L**) was rich in β-caryophyllene (22.2%) and caryophyllene oxide (12.4%), followed by 2-tridecanone (7.3%) and δ-cadinene (6.6%). However, the EO of the branches (**Cuj-B**) was dominated by 2-tridecanone (30.0%), limonene (20.5%), and β-caryophyllene (8.1%). The occurrence of fatty acid derivatives such as 2-tridecanone in Lauraceae species is not very common. However, β-caryophyllene, caryophyllene oxide, and δ-cadinene were the main compounds of the EO oil from leaves of *Cryptocarya mandioccana* [27].

The oils of *O. caniculata* showed predominance of sesquiterpenes of selinane and caryophyllane skeletons. In the leaves (**Can-L**), the main compounds were β-selinene (20.3%), β-caryophyllene (18.9%), 7-*epi*-α-selinene (14.3%), and bicyclogermacrene (10.4%); in the branches (**Can-B**), selin-11-en-4-α-ol (20.6%), β-selinene (12.1%), 7-*epi*-α-selinene (9.0%), β-caryophyllene (7.1%), and the phenylpropanoid (*E*)-asarone (8.8%) were identified. Interestingly, the leaf essential oil of *O. foetens*, an endemic species of the Macronesian region (Canary Islands, Spain, and Madeira Islands, Portugal), had very little concentration of monoterpenoids or sesquiterpenoids, but was rich in ethyl *p*-coumarate [28].

**Figure 2:**
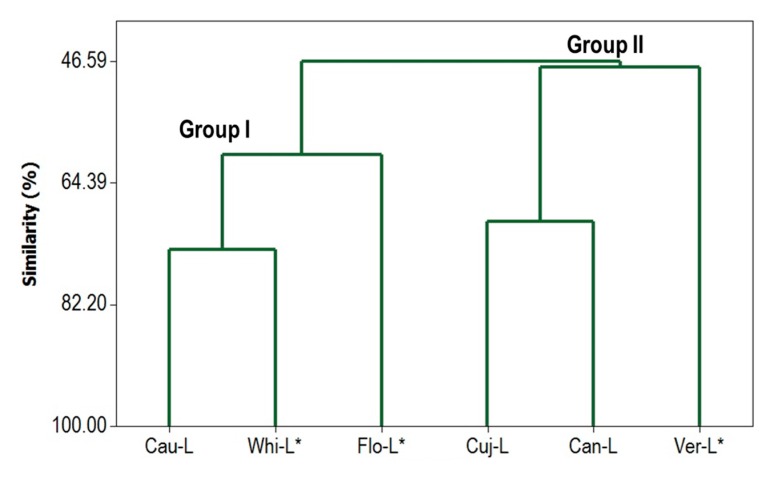
Dendrogram with Complete Linkage and Correlation Coefficient Distance. * Literature data (Takaku et al., 2007 [21]).

In order to differentiate between the analyzed *Ocotea* samples, a hierarchical cluster analysis using the chemical constituents was carried out and the resulting dendrogram is shown in Figure 2. According to these results, the samples were grouped in two main groups with similarity of 46.6%. Group I is characterized by oils from *O. caudata*, *O. whitei*, and *O. floribunda*, which present high amounts of α-pinene (9.8–22.5%) and β-pinene (7.3–21.3%). A higher similarity (74.1%) was found with *O. caudata* and *O. whitei* due the proportions of α-pinene, β-pinene, and β-caryophyllene (see Table 2). The main compound of *O. floribunda* oil was the diterpene kaurene (34.0%) and it decreased its similarity level. Group II includes *O. cujumary*, *O. caniculata*, and *O. veraguensis* oils, the samples **Cuj-L** and **Can-L** present similar amounts of β-caryophyllene (22.2% and 18.9%, respectively). A lower similarity was found in sample **Ver-L**, which was rich in bulnesol (29.0%) and *p*-cymene (19.8%).

### 2.2. Antimicrobial and Cytotoxic Activities

All essential oils displayed high antimicrobial activity against *E. coli* (minimum inhibitory concentration (MIC) < 19.5 μg·mL^−1^). **Cau-L** and **Cuj-L** oils showed notable activity against *S. epidermis* and *B. cereus* (Table 3). It is not obvious what component(s) are responsible for the activity against *E. coli*. Most essential oil components show only marginal activity against this organism. The antibacterial activities against the other bacteria are consistent with the activities of the essential oil components (Table 3). On the other hand, the cytotoxic activity against MCF-7 cells did not display variation in the median inhibitory concentration (IC_50_) values among all samples with an average of 63.0 μg·mL^−1^ (Table 3). The main compounds germacrene D, bicyclogermacrene, β-caryophyllene, α-pinene, β-pinene, caryophyllene oxide, β-selinene, 7-*epi*-α-selinene have been reported as antimicrobial and cytotoxic [29,30,31,32,33]. The observed cytotoxicities of some of the essential oil components are consistent with the cytotoxicities of the essential oils themselves (Table 3).

The EOs of leaves of *Endlicheria arenosa*, which are rich in bicyclogermacrene (42.2%), germacrene D (12.5%), and β-caryophyllene (10.1%), showed the same IC_50_ value against *Escherichia coli* [23]. The EO from the leaves of *Ruilopezia bracteosa* (Asteraceae), with higher amounts of α-pinene (24.3%), 7-*epi*-α-selinene (9.1%), and β-pinene (8.5%), showed antibacterial activity against Gram-positive and Gram-negative bacteria [34].

Terpenoids from plant oils prevent tumor cell proliferation through necrosis or induction of apoptosis [35]. Germacrene D is reported as cytotoxic against cervical carcinoma (HeLa), leukemia (HL-60), colon carcinoma (HCT), breast adenocarcinoma (SKBr), and melanoma (A2058) [36]. β-Caryophyllene and caryophyllene oxide displayed activity against several tumor cells such as breast cancer (MCF-7), colon cancer (HCT-116), and human prostate (PC-3) [37,38]. The values of IC_50_ of bicyclogermacrene, α-pinene, and β-pinene against MCF-7 cells were 19.0, 30.7, and 80.2 µg/mL, respectively [24,39].

### 2.3. Phylogenetic Analysis

*MatK* from chloroplast genes has been used as a marker for the construction of plant phylogenies, due to its rapid evolution and their ubiquitous presence in plants [40]. The sequences have a size of about 1550 bp and encode the maturase K enzyme [41].

**Figure 3:**
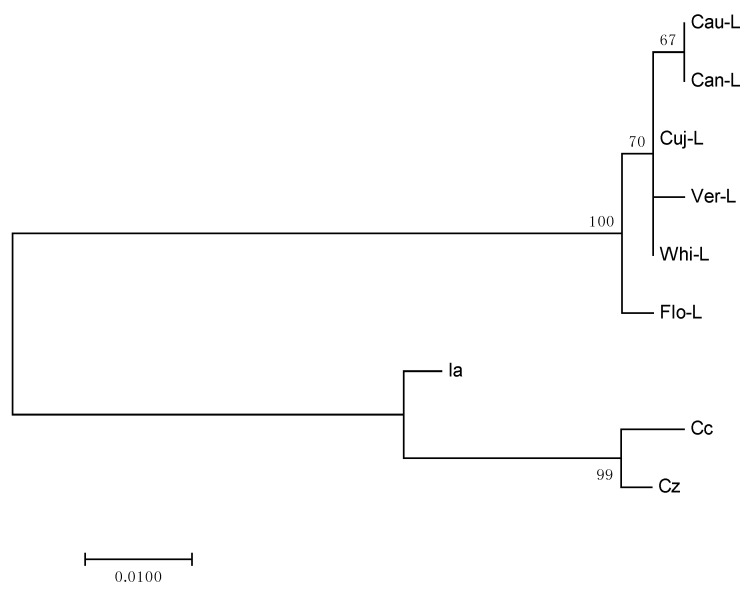
Molecular Phylogenetic analysis by Maximum Likelihood method for *matK* sequence of species of Lauraceae and Calycanthaceae (Outgroup).

Lauraceae species used in this study formed a defined group in the phylogenetic analysis, and the results of the phylogenetic tree were robust and supported by bootstrap value (=100). Species of Calycanthaceae were used as outgroup; this corroborates with the taxonomic classification and supports the topology of the tree (Figure 3).

The genetic distances estimated for Lauraceae species were lower. The values ranged from 0 to 0.003 to **Cau-L**, **Cuj-L**, **Can-L** and from 0 to 0.009 to **Ver-L**, **Flo-L** and **Whi-L**.

The tree shows that the geographically closest species are grouped: **Cau-L**, **Cuj-L**, and **Can-L** were collected in Caxiuanã National Forest and **Ver-L**, **Flo-L**, and **Whi-L** were collected in Monteverde, Costa Rica. Although there may be differences in the composition of essential oils in plants of the same species with the same geographical location, the results of phylogenetic tree showed a good correlation with classes of chemical compounds. The species **Cau-L** and **Can-L** are rich in sesquiterpene hydrocarbons and the samples **Cuj-L**, **Ver-L**, and **Whi-L** showed similar amounts of monoterpene hydrocarbons. A higher difference could be observed for sample **Flo-L**, which had diterpenes as its main compound class. Our results support the hypothesis that *matK* gene is reasonably useful in phylogenetic reconstructions at high taxonomic levels (to order or family), but shows poorer reliability with lower taxonomic levels of classification (to genus or species) [42,43].

## 3. Materials and Methods

### 3.1. Plant Material

Leaves and branches of *O. caudata* (Nees) Mez, *O. cujumary* Mart., and *O. caniculata* (Rich.) Mez were collected in Caxiuanã National Forest, Marajó Island, and their vouchers were deposited in the Herbarium of Museum Paraense Emílio Goeldi, Belém, Pará state, Brazil (Table 1).

### 3.2. Essential Oil Extraction

Leaves and branches were air-dried, pulverized, and subjected to hydrodistillation using a Clevenger-type apparatus (100 g, 3 h). The essential oils were dried over anhydrous sodium sulfate, and the essential oil yields were calculated on the basis of the dry weight of plant material. The moisture contents of the samples were calculated after phase separation using a Dean–Stark trap (5 g, 60 min) using toluene as the solvent phase.

### 3.3. Gas Chromatographic–Mass Spectral Analysis

The volatile compositions were analyzed by gas chromatography using an Agilent 6890 GC with an HP-5 ms column, and mass spectrometry with an Agilent 5973 mass selective detector (MSD) operated in the electron impact (EI) mode with electron energy = 70 eV. The scan range was 40–400 atomic mass units (amu) and the scan rate was 3.99 scans/s. The data were processed with an Agilent ChemStation data system. The gas chromatography (GC) column was a fused silica capillary with a (5% phenyl)-polymethylsiloxane stationary phase that had a film thickness of 0.25 μm and a length of 30 m, and an internal diameter of 0.25 mm. Helium was the carrier gas with a flow rate of 1.0 mL/min and a column head pressure of 48.7 kPa. The injector temperature was 200 °C and the detector temperature was 280 °C. The GC oven temperature was programed to start with an initial temperature of 40 °C, which was held for 10 min. The temperature was then increased at a rate of 3 °C/min to 200 °C, and then increased at 2 °C/min to 220 °C. A 1-μL injection of a solution (0.2% *w*/*v* in CH_2_Cl_2_) of the sample was performed using a splitless injection technique. The percentages of each component were based on total ion current and are reported without standardization. Individual components were identified by comparison of both their mass spectra and GC retention data with authentic compounds present in commercial libraries [44] and our own in-house library.

### 3.4. Antibacterial Assay

The antimicrobial activity of EOs was determined against *Bacillus cereus* (ATCC No. 14579), *Escherichia coli* (ATCC No. 10798), *Pseudomonas aeruginosa* (ATCC No. 27853), *Staphylococcus aureus* (ATCC No. 29213), and *Staphylococcus epidermidis* (ATCC No. 12228), using the microbroth serial dilution method as previously reported [45]. Thus, 50 μL of 1% *w*/*v* solution of the samples in dimethylsulfoxide (DMSO) was placed in the top well of 96-well microtiter plates and 50 μL of cation-adjusted Mueller Hinton broth (CAMHB) was added. The sample solutions were then serially diluted (1:1) by transferring 50 μL of sample-CAMHB mixture to the next lane and adding 50 μL fresh CAMHB to obtain a concentration range from 2500 to 12.5 μg·mL^−1^. The bacteria were harvested from a fresh culture and added to each well at a concentration of approximately 1.5 × 10^8^ colony forming units (CFU)·mL^−1^. The plates were incubated at 37 °C for 24 h and the final minimum inhibitory concentrations (MICs) were determined as the lowest concentrations free of turbidity. The antibiotic gentamicin was used as positive control and DMSO solvent was used as negative control.

### 3.5. Cytotoxicity Assay

MCF-7 human mammary adenocarcinoma cells (ATCC No. HTB-22) were cultured in RPMI (Roswell Park Memorial Institute) 1640 medium supplemented with 10% fetal bovine serum (FBS), 30 mM HEPES (4-(2-hydroxyethyl)-1-piperazineethanesulfonic acid buffer), NaHCO_3_, and penicillin streptomycin. The cytotoxicity of the essential oils on MCF-7 cells was determined using the 96-well MTT (3-(4,5-dimethylthiazol-2-yl)-2,5-diphenyltetrazolium bromide) assay as reported previously [45]. Cells were seeded into 96-well cell culture plates at a concentration of 1.2 × 10^4^ cells/well and a volume of 100 μL in each well. The plate was then labeled and incubated at 37 °C and 5% CO_2_ for 48 h. After 48 h, the cells reached 70–80% confluent growth. The supernatant fluid was gently aspirated (without touching the bottom of the well to avoid removing cells) and replaced with 100 μL growth medium that contained either 1.0 or 0.5 μL of essential oil (1% in DMSO), to give final concentrations of 100 and 50 μg·mL^−1^. The plate was then incubated at 37 °C and 5% CO_2_ for 48 h. After 48 h, the liquid was gently aspirated from each well. In a tube, 10 mL feeding medium was mixed with 2 mL of MTT stock solution (and was protected from light). Into each well, 100 µL of the MTT solution was added and the pre-read absorbance was immediately measured spectrophotometrically at 570 nm (using a Molecular Devices Spectra Max Plus 384 microplate reader). Formazan crystals were formed over the course of 4 h at 37 °C and 5% CO_2_. After 4 h, DMSO was used to dissolve the purple crystals. The amount of MTT-formazan produced was determined spectrophotometrically at 570 nm. Growing medium, DMSO, and tingenone (100 μg·mL^−1^) served as negative, compound, and positive controls, respectively. Solutions were added to wells in eight replicates. Average absorbances, standard deviations, and percent kill ratios (% kill_oil_/% kill_control_) were calculated. Median inhibitory concentrations (IC_50_) were determined using the Reed-Muench method [46].

### 3.6. Multivariate Statistical Analysis of Chemical Composition

Hierarchical cluster analysis was carried out to organize and cluster the essential oils according to their main volatile constituents. Complete linkage and absolute correlation coefficient distance was chosen to determine similarity. Agglomerative hierarchical clustering was utilized for clustering the essential oils. The MINITAB 14.0 software was used to statistically analyze all data.

### 3.7. DNA Isolation, PCR Amplification, and Sequencing

Genomic DNA material was extracted from dried leaf tissue of each plant using E.Z.N.A^®^ Poly-Gel DNA Extraction Kit (Omega Bio-tek, Norcross, GA, USA) according to the protocol given by the company. For amplification of *matK* region, PCR was performed in a 50 µL reaction mixture that consisted of 36.5 µL of nanofiltrated (NF) water, 5.0 µL of 10× Advantage Buffer, 1.0 µL of deoxynucleotide (DNTP), 0.5 µL of Taq-DNA polymerase, 5.0 µL template DNA, 1.0 µL of *matK*-Lau001 (F-5’-TCCTTTCTTGAGCGAACACA-3’), and 1.0 µL of *matK*-Lau002 (R-5’-CTGACAAATCGGACCGAAAC-3’) primers purchased from Eurofins MWG Operon (Huntsville, AL, USA). Amplifications conditions for *matK* primers consisted of an initial denaturation step for 2 min at 95 °C, followed by 40 cycles of 30 s at 95 °C, 30 s at 61 °C, 60 s at 68 °C, and 60 s at 72 °C for final extension. Amplifications were performed in an Eppendorf MasterCycler Pro thermocycler, the PCR products were visualized in agarose gel (1%), purified, and then they were sent to Eurofins MWG Operon (Huntsville, AL, USA) for sequencing.

### 3.8. Phylogenetic Analysis

The *matK* sequences of *O. caudata* (Nees) Mez, *O. cujumary* Mart., and *O. caniculata* (Rich.) were assembled and edited using FinchTV (biomatters Ltd., Auckland, New Zealand). Sequences were checked against those in GenBank using the BLAST algorithm. The *matK* sequences from *O. caudata* (Nees) Mez, *O. cujumary* Mart., and *O. caniculata* (Rich.) were aligned with sequences from *O. floribunda* (gbEU153866.1), *O. veraguensis* (gb.JQ589849.1), and *O. whitei* (gb. JQ589831.1) available in GenBank. The sequences were edited using BioEdit V7.2.5 [47] and the alignment was performed using the Clustal W [48]. The distance genetic and Phylogenetic Tree was determined using Mega 7 Program [49]. The evolutionary history was inferred using the maximum likelihood method and the evolutionary distances were computed using the Kimura 2-parameter model with 10,000 replicates. The outgroups used for the construct phylogenetic tree were the *matK* sequences from *Calycanthus chinensis* (Cc) (gb|AY525339.1), *Idiospermum australiense* (Ia) (gi71891392), and *Chimonanthus zhejiangensis* (Cz) (gb|AY525341.1) belonging to the order Laurales and Calycanthaceae family.

## 4. Conclusions

In this present study, the main compounds identified in the essential oils of species collected in Caxiuanã Forest were terpenoid hydrocarbons and oxygenated terpenoids, which had good antimicrobial and cytotoxic activities. The main class of chemical compounds displayed a good correlation with chloroplast DNA region (*matK* gene) analysis. However, the analysis of phylogenetic data and specific chemical compounds showed differences. These results suggest that *matK* gene was not efficient for representing this relationship.

## Figures and Tables

**Table 1:** Collection data and essential oil yield of the samples of *Ocotea* occurring in Caxiuanã National Forest, Amazon, Brazil.

Species	Geographic Coordinate	Voucher	Plant Material	Sample	Oil Yield (%)
*O. caudata*	S 01.0° 44.0′ 18.8′′	MG 216263	Leaves	Cau-L	0.7
	W 51.0° 27.0′ 27.4′′		Branches	Cau-B	0.1
*O. cujumary*	S 01.0° 44.0′ 14.1′′	MG 216269	Leaves	Cuj-L	0.8
	W 51.0° 27.0′ 20.4′′		Branches	Cuj-B	0.5
*O. caniculata*	S 01.0° 44.0′ 14.1′′	MG 216262	Leaves	Can-L	0.7
	W 51.0° 27.0′ 20.4′′		Branches	Can-B	0.2

**Table 2:** Chemical composition of essential oils of *Ocotea* species. RI^Calc^, calculated retention index; RI^Lit^, literature retention index.

Constituents	RI^Calc^	RI^Lit^	Cau-L	Cau-B	Cuj-L	Cuj-B	Can-L	Can-B	Flo-L *	Ver-L *	Whi-L *
*E*-2-Hexenal	856	854								1.1	0.8
α-Thujene	933	931								0.2	
α-Pinene	936	932	9.8		2.1				22.5	0.7	12.7
Camphene	956	953							1.7	0.1	0.3
Sabinene	979	976								0.1	
β-Pinene	980	974	9.7		1.8	2.2			21.3	0.3	7.3
Myrcene	995	991							0.7	1.1	0.5
α-Phellandrene	1009	1005								1	
*p*-Cymene	1028	1026								19.8	
Limonene	1030	1024	2.1		1.8	20.5			2.7		1.1
β-Phellandrene	1032	1031								4.0	
1,8-Cineole	1033	1033									1.3
γ-Terpinene	1064	1062									0.1
α-Terpinolene	1089	1088									0.1
α-Pinene oxide	1097	1095							0.1		
Linalool	1104	1098								1.7	
Borneol	1168	1165								0.1	
Terpinen-4-ol	1178	1177						2.0		0.2	
α-Terpineol	1191	1189								0.2	1.3
Cuminal	1238	1239								0.1	
2-Undecanol	1301	1301			1.2	4.6					
δ-Elemene	1340	1335	2.2								
α-Cubebene	1351	1345	2.0	2.0	0.8	2.2					
α-Ylangene	1373	1373	0.8		5.1						
α-Copaene	1377	1374	1.0						0.1	0.1	0.8
β-Cubebene	1392	1387	1.6						0.1		
β-Bourbonene	1384	1387			0.7					0.1	0.1
δ-Elemene	1392	1389	1.5		0.4		1.1	0.8	0.3	0.7	0.3
*Z*-Caryophyllene	1416	1408			0.3						
β-Caryophyllene	1421	1417	9.6	2.5	22.2	8.1	18.9	7.1	2.5	2.3	15.2
2,5-Dimethoxy-*p*-cymene	1424	1424						0.9			
β-Copaene	1428	1430			0.4						
β-Gurjunene	1432	1432									0.2
α-*trans*-Bergamotene	1436	1432					0.9				1.9
γ-Elemene	1435	1434	0.8								0.1
α-Guaiene	1439	1439							0.1		
Aromadendrene	1440	1439									0.1
*Z*-β-Farnesene	1443	1440						0.9			
Spirolepechinene	1451	1449	0.7				1.4	0.6			
α-Humulene	1455	1452	1.8	2.4	3.8	2.5	2.5	1.7	0.3	1.7	1.7
Sesquisabinene	1458	1457						0.9			
Dehydroaromadendrane	1460	1460					0.6				
*E*-β-Farnesene	1462	1458									0.9
*allo*-Aromadendrene	1463	1461									0.2
*trans*-Cadina-1(6),4-diene	1473	1475			0.5						
γ-Selinene	1477	1470								0.2	
γ-Gurjunene	1477	1475						0.4			
γ-Muurolene	1476	1478			0.8			0.7			
Widdra-2,4(14)-diene	1483	1481		6.5							
Germacrene D	1484	1484	19.9	8.9	0.9	5.9			1.2	0.2	5.5
β-Selinene	1485	1489			2.2		20.3	12.1		0.3	
Valencene	1493	1491								1.1	
*cis*-β-Guaiene	1493	1492		8.3	3.0			5.2			
*trans*-Muurola-4(14),5-diene	1491	1493			1.1						
2-Tridecanone	1497	1495			7.3	30.0					
Viridiflorene	1493	1496					9.8				
Bicyclogermacrene	1500	1500	29.6				10.4				5.3
α-Muurolene	1499	1500		1.7				1.0	0.1		
Germacrene A	1504	1503								0.2	2
β-Bisabolene	1509	1505			0.6						
δ-Amorphene	1509	1511	0.8	2.0							
*E*,*E*-α-Farnesene	1510	1508									0.3
γ-Cadinene	1517	1513	0.9	5.9	1.6	3.1		1.7	0.1	0.4	0.7
7-*epi*-α-Selinene	1517	1520		4.5			14.8	9.0			
β-Cadinene	1519	1518								0.7	0.6
*cis*-Calamenene	1523	1521								0.6	
δ-Cadinene	1526	1522	1.4	13.8	6.6	4.7	0.6	3.9	0.2	0.4	3.7
*trans*-Cadina-1,4-diene	1532	1533			0.6					0.2	0.2
α-Cadinene	1537	1538									0.1
α-Calacorene	1542	1544			0.9			1.5		0.2	
Elemol	1549	1549									0.1
Germacrene B	1558	1559	1.6	3.3			0.8	0.4			0.7
*E*-Nerolidol	1564	1561					1.4			2.5	3.9
β-Calacorene	1564	1564						0.7		0.5	
γ-Asarone	1576	1572						0.4			
Spathulenol	1579	1577	1.4		0.5		1.0		6	8.5	15.3
Caryophyllene oxide	1585	1582	1.0	2.0	12.4		0.9	2.0			
Globulol	1592	1590						0.9			
Viridiflorol	1597	1592						1.2			
Carotol	1586	1594		2.7							
6-Methoxyelemicin	1601	1595						6.3			
Guaiol	1597	1600			1.2	4.0				5.2	
Humulene epoxide II	1608	1608			1.0					1.7	
*Z*-Asarone	1625	1616					3.4				
1,10-di-*epi*-Cubenol	1620	1618		3.0				1.4			
Junenol	1620	1618						0.6			
1-*epi*-Cubenol	1628	1627		3.8				0.4		3.3	
Muurola-4,10(14)-dien-1β-ol	1628	1630			3.3						
*epi*-α-Cadinol	1642	1638						2.0		0.5	
*allo*-Aromadendrene epoxide	1632	1639			1.4						
Caryophylla-4(12),8(13)-dien-5β-ol	1636	1639			3.2						
α-Muurolol	1642	1644		7.8					0.9	1.8	
Cubenol	1646	1645		2.2	1.5						
α-Cadinol	1652	1652		2.2					1.8	1.5	1.8
Valerianol	1654	1656		6.8							
Selin-11-en-4α-ol	1657	1658		3.1				20.6			
7-*epi*-α-Eudesmol	1658	1662					4.2				
Bulnesol	1662	1666								29.5	
14-Hydroxy-(*Z*)-caryophyllene	1666	1666		2.3	1.5		0.9	2.1			
*E*-Asarone	1683	1675					3.6	8.8			
β-Sinensal	1699	1699									0.6
*E*,*E*-Farnesylacetate	1843	1843									10.1
Isohibaene	1922	1923							0.7		
Kaurene	2034	2034							34.0		

RI^Calc^ = based on DB-5ms capillary column and alkane standards (C8-C32). RI^Lit^ = based on Adams library. * Literature data (Takaku et al., 2007 [22]).

**Table 3:** Antimicrobial and cytotoxic activities of *Ocotea* essential oils and some major essential oil components. MIC, minimum inhibitory concentration; IC_50_, median inhibitory concentration.

Material	MIC (μg·mL^−1^)					IC_50_ (μg·mL^−1^)
	*P. aer*	*E. coli*	*S. epi*	*S. aur*	*B. cer*	MCF-7
**Can-L**	1250.0	19.5	625.0	625.0	625.0	63.9 ± 3.7
**Cau-L**	1250.0	19.5	625.0	625.0	312.5	64.0 ± 3.7
**Cuj-L**	1250.0	19.5	312.5	625.0	312.5	67.7 ± 3.7
**α-Pinene**	625	312	-	312	625	69.5 ± 1.3
**β-Pinene**	1250	625	-	625	312	71.2 ± 2.0
**Limonene**	1250	625	-	312	625	77.4 ± 1.2
**β-Caryophyllene**	1250	312	-	312	156	59.4 ± 5.1
**α-Humulene**	1250	625	-	312	312	26.5 ± 5.6
**Germacrene D**	1250	625	-	156	625	69.6 ± 2.5
**Caryophyllene oxide**	1250	1250	-	1250	156	73.4 ± 3.7
**Gentamicin control**	1.22	2.44	<19.5	0.61	1.22	-
**Tingenone control**	<19.5	<19.5	-	2.44	1.22	16.8 ± 1.7

*P. aer* (*Pseudomonas aeruginosa*), *E. coli* (*Escherichia coli*), *S. epi* (*Staphylococcus epidermidis*), *S. aur* (*Staphylococcus aureus*), *B. cer* (*Bacillus cereus*).

## Abbreviations

matK	Megakaryocyte-Associated Tyrosine Kinase
MIC	Minimum Inhibitory Concentration
RI_Calc._	Calculated retention index
RI_Lit_	Literature retention index
IC_50_	Median Inhibitory Concentration
GC	Gas chromatography
DMSO	Dimethylsulfoxide
RPMI	Roswell Park Memorial Institute medium
MTT	3-(4,5-Dimethylthiazol-2-yl)-2,5-diphenyltetrazolium bromide
HEPES	4-(2-Hydroxyethyl)-1-piperazineethanesulfonic acid buffer
PCR	Polymerase Chain Reaction
